# Gas Quenching under Ambient Conditions for Efficient and Stable Wide-Bandgap Perovskite Solar Cells with Surface Passivation

**DOI:** 10.1021/acsami.5c21175

**Published:** 2025-12-31

**Authors:** Zhaonan Jin, Xiongzhuo Jiang, Zerui Li, Xiaojing Ci, Guangjiu Pan, Lixing Li, Jinsheng Zhang, Xinyu Jiang, Sarathlal Koyiloth Vayalil, Kun Sun, Stephan V. Roth, Peter Müller-Buschbaum

**Affiliations:** † TUM School of Natural Sciences, Department of Physics, 9184 Technical University of Munich, Chair for Functional Materials, James-Franck-Str. 1, 85748 Garching, Germany; ‡ 28332 Deutsches Elektronen-Synchrotron DESY, Notkestrasse 85, 22607 Hamburg, Germany; § Department of Physics, Applied Science Cluster, UPES, 248007 Dehradun, India; ∥ Department Perovskite Tandem Solar Cells, 28340 Helmholtz-Zentrum Berlin für Materialien und Energie GmbH (HZB), Kekuléstr. 5, 12489 Berlin, Germany; ⊥ Department of Fibre and Polymer Technology, KTH Royal Institute of Technology, Teknikringen 56-58, 11428 Stockholm, Sweden

**Keywords:** perovskite solar cells, gas quenching, 2D perovskite, surface passivation, organic spacer cation

## Abstract

Wide-bandgap perovskite solar cells play a key role in tandem solar cells, which aim to overcome the Shockley–Queisser limit for single-junction solar cells. In this work, we develop and optimize a gas quenching method under ambient conditions for the fabrication of wide-bandgap (1.77 eV) perovskite films. To improve the performance of PSCs, three different organic spacer cations, including aromatic amino molecules (PEAI), aliphatic amino with long alkyl chain molecules (OAI), and short alkyl chain molecules (BAI), are applied and investigated as surface passivation materials. As a result, the 2D perovskite layers form on top of the 3D perovskite films. The n-i-p devices with PEAI passivation exhibit the highest photovoltaic performance with a champion power conversion efficiency (PCE) of 16.26% along with a high *V*_oc_ of 1.21 V, exceeding the control device (PCE = 13.42%, *V*_oc_ = 1.15 V), and maintaining 88% of its initial PCE after 120 min of continuous illumination under a nitrogen atmosphere at room temperature. This work offers a guide for the fabrication of wide-bandgap PSCs under ambient conditions and the choice of organic spacer cations for passivation.

## Graphical abstract

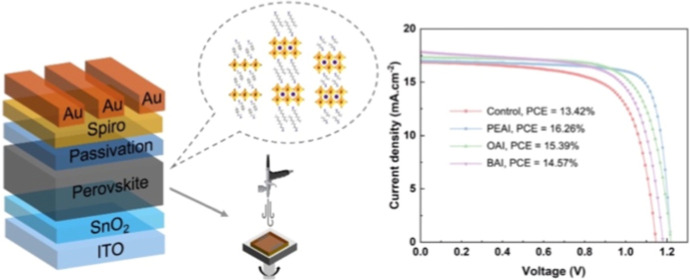

## 1 Introduction

The growing demand for renewable energy has accelerated the development of photovoltaic technologies. In this context, perovskite solar cells have rapidly emerged as one of the most promising photovoltaic technologies in the last years, exhibiting a rapid progress in power conversion efficiency (PCE) values from less than 4% to over 26%.^1,2^ Compared to traditional silicon solar cells, perovskite materials have the merits of strong light absorption, high charge carrier mobilities, long charge carrier diffusion lengths, tunable bandgaps, and high defect tolerance.^3,4^ The optical bandgap of perovskite materials can be tuned over a wide range (∼1.2–3.0 eV) by substituting the halide composition (e.g., I^–^–Br^–^–Cl^–^) or modifying the A-site cation to influence crystal symmetry and lattice parameters.^3–5^ In particular, wide-bandgap perovskites (WBG, *E*_*g*_ ∼ 1.7–1.9 eV) have been extensively explored as the top cell absorber in monolithic two-terminal or mechanically stacked four-terminal tandem architectures. Their high open-circuit voltage and ability to absorb a distinct spectral range make them ideal for pairing with low bandgap materials such as silicon, organic, CIGS, or narrow-bandgap perovskites to surpass the Shockley–Queisser limit of single-junction solar cells.^5–7^

The crystallization kinetics of perovskite films play a decisive role in determining the film morphology, device performance, and device stability.^8–10^ Among the various methods for controlling the crystallization process, the antisolvent method—where a nonpolar solvent is dripped onto the wet film to rapidly induce supersaturation—requires precise timing and solvent compatibility with the precursor system. However, it remains largely limited to laboratory-scale fabrication and poses significant challenges for upscaling.^11–13^ In contrast, gas quenching has been demonstrated to be versatile in various coating techniques.^14–17^ It uses a stream of inert gas (typically nitrogen or dry air) directed onto the wet film during the coating process. The gas flow accelerates solvent evaporation uniformly across the substrate, leading to a controlled supersaturation and crystallization without introducing an antisolvent. Huang et al. introduced a gas quenching technique, where a stream of gas was directed at the substrate during the spin coating of a DMF-based precursor.^14^ This method led to the formation of a smooth MAPbI_3_ film and yielded average PCEs of around 16%.^14^ Building on this concept, Conings et al. explored a gas quenching solution deposition approach assisted by DMSO–PbX_2_ complex formation. This approach enabled the fabrication of pinhole-free FA_0_._8_Cs_0_._2_Pb­(I_0_._65_Br_0_._35_)_3_ perovskite films (with a bandgap of 1.76 eV), resulting in devices with a champion PCE of 13%.^15^ This gas quenching method offers significant advantages in terms of film uniformity, process reproducibility, and environmental adaptability. Given these strengths, gas quenching demonstrates strong potential for widespread application and industrial scalability, accelerating the transition of perovskite solar cell technology from laboratory research to commercial deployment.

Nevertheless, the formation of defects in the perovskite film remains possible under practical experimental gas quenching conditions. The performance of perovskite solar cells is influenced by a variety of factors, with the most critical being the quality of the perovskite film such as its degree of crystallization, grain size, uniformity, and the presence of defects. In addition, the interfacial quality between the perovskite layer and the charge transport layers significantly affects the charge carrier extraction and transport efficiency, thereby impacting the overall performance of the PSCs. Surface passivation of perovskite films is a crucial strategy to enhance the performance and stability of PSCs by reducing surface defects and nonradiative recombination. This strategy involves introducing specific materials or molecules, such as an organic amine,^18,19^ polymer,^20^ Lewis acid/base,^21^ inorganic molecule,^22^ and low-dimensional perovskite,^23,24^ that can effectively interact with defects and improve electronic properties of the perovskite films. Among these, two-dimensional (2D) Ruddlesden–Popper (RP) phase perovskites, described by the general formula L_2_A_*n*–1_B_*n*_X_3*n*+1_ (where L is an organic spacer cation, A is a monovalent organic cation, B is a divalent metal cation, X is a halide ion, and *n* indicates the number of perovskite octahedral layers), have attracted significant attention.^25,26^ The incorporation of large organic spacer cations results in quantum well structures, where the inorganic perovskite layers are encapsulated by insulating organic layers.^27^ Chen et al. used a phenethylammonium iodide-based (PEAI-based) surface treatment to construct 2D RP phase perovskite layers with different *n* values on 3D perovskite films for passivation, and systematically explored the degradation mechanisms of the induced 2D perovskites with different *n* values.^28^ Several studies have investigated how organic spacer cations with different numbers of aromatic rings and varying alkyl chain lengths influence the interfacial properties of the perovskite thin films, while the induced 2D perovskites were not probed in these studies. Xiang et al. explored the impact of surface passivation using aromatic amino cations of PEAI and naphthylethylammonium iodide (NEAI) with varying numbers of benzene rings on the structural and optoelectronic properties of the perovskite films, and NEAI was revealed to not only improve the perovskite layer/HTL interface but also make the modified films more stable against moisture due to the better hydrophobicity.^29^ Kim et al. compared the influence of different lengths of alkylammonium halides, like butylammonium iodide (BAI), octylammonium iodide (OAI), and dodecylammonium iodide (DAI), on the device performance as surface passivation. The results showed that the device with OAI passivation achieved a higher and more stable PCE.^30^ In a word, the 2D perovskite as a passivation layer is regarded as an efficient method to improve the efficiency and stability of PSCs.

In this study, we develop a gas quenching method under ambient conditions and optimize the experimental parameters, such as different spin coating speeds and gas flow pressure, to fabricate 1.77 eV wide-bandgap 3D perovskite films. For the further enhancement of the PSCs, organic spacer cations with varying molecular structures, specifically aromatic amino molecules (PEAI), aliphatic amino with long alkyl chain molecules (OAI), and short alkyl chain molecules (BAI), are applied and investigated as surface passivation materials. Therefore, 2D perovskite layers form on top of the 3D perovskite films and subsequently influence the interfacial properties and device performance of the PSCs. As a result, the champion device with PEAI passivation exhibits an enhanced *V*_oc_ of 1.21 V and a PCE of 16.26%, exceeding the control device (PCE = 13.42%, *V*_oc_ = 1.15 V) and maintaining 88% of its initial PCE after 120 min of continuous illumination under a nitrogen atmosphere at room temperature. This study provides a practical solution to fabricate wide-bandgap PSCs under ambient conditions and to screen the applicable organic spacer cations for surface passivation, showing great potential for low-cost, effective, and scalable production of wide-bandgap PSCs used in tandem solar cells.

## 2 Results and Discussions

### 2.1 Optimization of Gas Quenching

The quality of the perovskite film directly affects the performance of the solar cells. High-quality films with good crystallinity, low defect density, and uniform coverage enable efficient charge carrier transport and reduced nonradiative recombination losses, resulting in higher open-circuit voltages, short-circuit currents, and overall improved PCEs. In contrast, poor-quality films with many defects or pinholes can cause charge carrier recombination and leakage, significantly limiting device performance.

Gas quenching and conventional antisolvent methods exhibit distinct differences in perovskite film formation: gas quenching rapidly removes solvent and instantaneously promotes crystallization, resulting in uniform crystals, dense films, and smooth surfaces, whereas conventional antisolvent methods rely on timed solvent dripping to induce rapid crystallization,^31^ which is highly sensitive to timing and environmental conditions and prone to wrinkles, needle-like crystals, pinholes, and nonuniform surfaces, leading to lower film density and uniformity, higher operational difficulty, and limited scalability.^32^ Therefore, gas quenching is an effective method to control the crystallization behavior and improve film quality. The process of gas quenching as applied in this study is shown in Figure 1a. Parameters such as nozzle distance, quenching timing, spin coating speed, and gas flow pressure significantly affect the film crystallization, morphology, and defect density. In this study, we primarily investigate how the spin coating speed and gas flow pressure affect the perovskite film properties. A scanning electron microscope (SEM) and an atomic force microscope (AFM) are used to characterize the surface morphology of pristine perovskite films fabricated under different conditions, as presented in Figures S1 and S2. With the spin coating speed increasing from 2000 to 4000 rpm and the gas flow pressure fixed at 2 bar, the surface perovskite grain size and film roughness decrease, and voids begin to emerge in the sample fabricated at the highest speed, according to Figure S1. A faster spin coating speed accelerates the evaporation of the solvent in the precursor solution and promotes rapid nucleation of perovskite crystals; however, it concurrently limits the time available for crystal growth, resulting in discontinuous films and increased defect formation.^33^ X-ray diffraction (XRD) is used to systematically investigate the crystallinity and phase composition of the pristine perovskite films fabricated under varying spin coating speeds (2000, 3000, and 4000 rpm) and gas flow pressures (2, 3, and 4 bar). As seen in Figure 1b,c, the peak intensity of residual PbI_2_ increases in the XRD spectra due to the rapid evaporation of solvent and an incomplete crystallization as the spin coating speed and gas pressure increase, which may hinder the charge carrier transport and accelerate degradation.^34^ The increased intensity of the PbI_2_ XRD peaks under higher spin-coating speeds and gas pressures is a direct consequence of the extremely rapid solvent evaporation and supersaturation induced by the gas-quenching process.^35^ This accelerated kinetics leads to a burst of nucleation but severely reduces the timeline for the full conversion of intermediate phases (e.g., DMSO–PbI_2_ complexes) into the phase-pure perovskite and complete grain growth.^36^ Consequently, unreacted PbI_2_ precursors are kinetically “frozen” within the film, manifesting as enhanced residual PbI_2_ diffraction signals. According to Figure S2, with an increasing gas flow pressure at a fixed spin coating speed of 2000 rpm, the surface grain size remains unchanged, but the roughness decreases. This finding can be attributed to the increased surface content of PbI_2_ shown in Figure 1c, providing a certain degree of passivation to the film. Therefore, the optimal gas quenching parameters in this work are determined as a 2000 rpm spin coating speed and 2 bar gas flow pressure. To quantify the impact of different spin-coating speeds and gas quenching conditions on the final solar cell performance, current density–voltage (*J*–*V*) measurements are conducted on unpassivated control devices fabricated with those conditions. As shown in Figure S3, the highest performance (average PCE of 12.8%, champion *V*_oc_ of 1.12 V) is achieved in devices fabricated under 2000 rpm and 2 bar conditions, which aligns perfectly with the optimal processing window identified from the prior analysis of film morphology and crystallinity. The pronounced performance disparities confirm that deviations from these optimal parameters lead to irreversible losses in the device efficiency.

**Figure 1:**
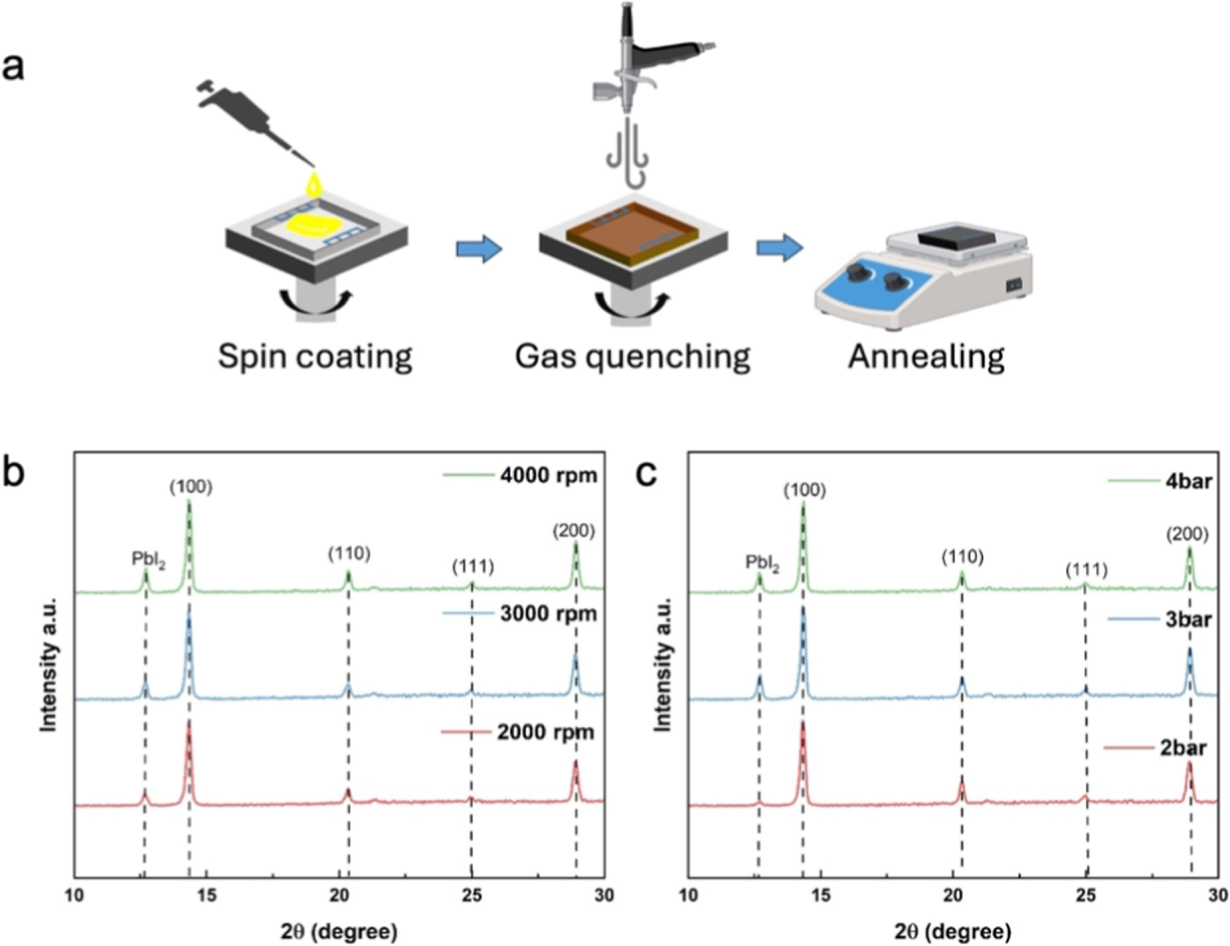
(a) Schematic drawing of the gas quenching process applied during film fabrication via spin-coating. XRD spectra of perovskite films fabricated by gas quenching with (b) different spin coating speeds (2000 rpm, 3000 rpm, 4000 rpm) at a fixed gas flow pressure of 2 bar, and (c) different gas flow pressures (2 bar, 3 bar, 4 bar) at a fixed spin coating speed of 2000 rpm.

### 2.2 Effect of Organic Spacer Cations on a Perovskite Film

Previous research has demonstrated that such 2D perovskite passivation layers can significantly impact the film quality by modifying the surface morphology, grain size, and overall roughness.^37,38^ Also, it has been observed that during solution-based processing, the introduction of 2D perovskites can induce recrystallization of the original surface grains, thereby altering the grain size.^39^ Therefore, we screen three representative organic spacer cations, namely, PEA^+^, BA^+^, and OA^+^, and investigate their effects on the surface morphology and photophysical properties of perovskite films. The molecular structures of them are presented in Figure 2a. After surface passivation by PEAI, OAI, and BAI, a clear surface morphology change is observed by SEM as shown in Figure 2b, revealing that the control perovskite film without surface passivation exhibits noticeable pinholes, whereas the passivated films display fewer defects. However, it can be observed that the passivation treatment on the surface has a minimal effect on the grain size, as both pristine and passivated films exhibit a maximum grain size of approximately 1 μm, consistent with Liu’s work.^40^ The minimal change in grain size may be attributed to the fact that the grain size tends to be fixed after annealing, followed by spin-coating and gas quenching, preventing further recrystallization. This trend aligns with the finding of Wu et al. that the grain size tends to stabilize following the deposition of the antisolvent.^23^ The roughness of the perovskite film surfaces reduces from 27.4 nm to around 20.0 nm after surface passivation, as shown in Figure 2c, forming a smoother surface that promotes efficient charge carrier extraction and suppresses ion movement.

**Figure 2:**
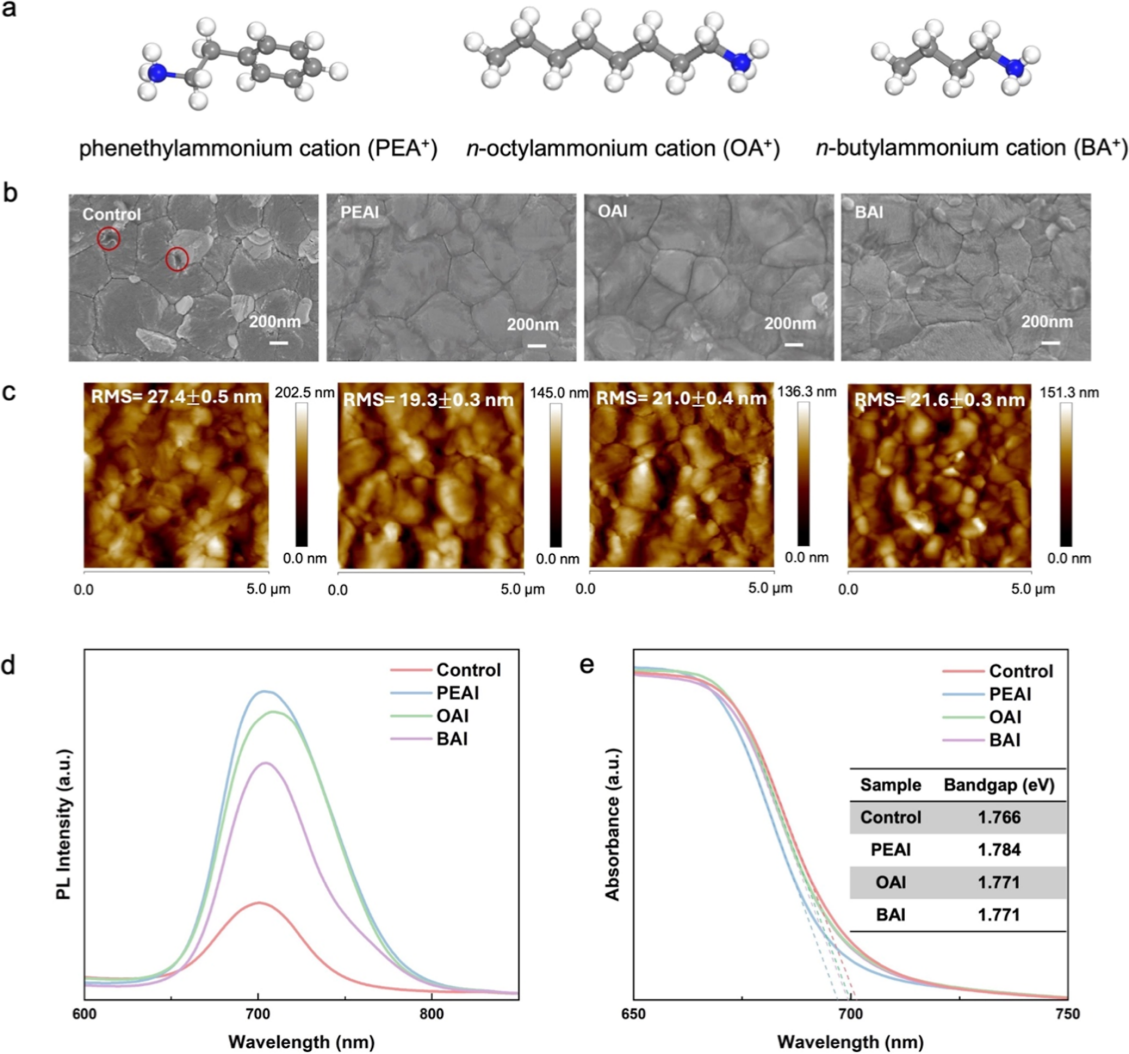
(a) Molecular structures of the three surface passivation materials used. (b) Top-view SEM images, (c) AFM topography images, (d) steady photoluminescence (PL) spectra, and (e) UV–vis optical absorption spectra of pristine perovskite film without surface passivation and films with PEAI, OAI, and BAI passivation. Surface defects are marked by red circles.

Interface defect-induced nonradiative recombination is a crucial factor that limits the open-circuit voltage in PSCs. PL measurements of perovskite films with and without surface passivation are performed to investigate this. As illustrated in Figure 2d, all samples exhibit a characteristic emission peak of FA_0.8_Cs_0.2_Pb­(I_0.6_Br_0.4_)_3_ perovskite around 700 nm. Among them, the emission peak of the OAI sample shows a slight redshift. This could result from the longer OAI organic molecules tending to adopt a preferential orientation on the surface and form a more ordered interfacial dipole layer, causing a downward shift in the surface energy levels and consequently leading to the PL redshift. Notably, the passivated samples display higher PL intensities compared with the Control sample, with the PEAI sample showing the strongest emission. The enhanced PL intensity is attributed to the improved film quality and reduced defect density at the perovskite surface, which decreases the number of charge carriers available for nonradiative recombination.^41^ The perovskite films with surface passivation exhibit wider bandgaps compared to the nonpassivated film, as determined by UV–vis absorption spectra shown in Figure 2e. The Control sample exhibits a bandgap of ∼1.77 eV, in good agreement with the literature bandgap value of FA_0_._8_Cs_0_._2_Pb­(I_0_._6_Br_0_._4_)_3_ perovskite.^42^ All four samples show bandgap values of 1.76 eV–1.79 eV, which is within the desired bandgap range (1.5 eV–2.2 eV) of the top perovskite cell in tandem solar cells.^43^ According to the following equation: $V_{oc}=\frac{E_{g}}{q}-V_{oc,loss}$ , such an increase in bandgap after passivation is expected to result in a higher open-circuit voltage (*V*_oc_) for the passivated samples.^44^

Grazing incidence wide-angle X-ray scattering (GIWAXS) analysis is a key tool for characterizing the crystal structure and orientation of perovskite films. It is valuable for providing detailed information on the layered structure and preferential orientation of the 2D and 3D perovskite phases. In the GIWAXS measurements, 0.1–0.6° increments in 0.1° increments are used as incident angles to perform angular-dependent measurements on perovskite films with and without surface passivation. The angular-dependent GIWAXS provides depth-dependent structural information due to variation in the X-ray probing depth. At lower incidence angles, GIWAXS primarily probes the near-surface region of the perovskite film, whereas at higher incidence angles, the structural information from deeper-lying structures within the bulk of the perovskite film is detected. According to the 2D GIWAXS data and pseudo XRD spectra shown in Figures 3a–c and S3, all four perovskite samples exhibit distinct Debye–Scherrer diffraction rings at *q* = 1.00, 1.40, 1.70, and 2.00 Å^–1^, corresponding to (100), (110), (111), and (200) 3D perovskite diffraction peaks, respectively, indicating the isotropic orientation of the 3D perovskite crystallites. In all passivated samples, the distinct 2D perovskite diffraction peaks observed in the GIWAXS data suggest a high face-on orientation of the crystalline domains, as illustrated in Figure S4b–d. For the PEAI sample, a dominant 2D perovskite (abbreviated as 2D PVK) diffraction peak emerges with decreasing incident angle at a *q* value of 0.36 Å^–1^, shown in Figure 3a, which is indexed as the (020) plane (*n* = 1) of the layered 2D RP phase perovskite crystals. In addition, 2D perovskite diffraction peaks are observed at *q* = 0.26 Å^–1^ (*n* = 2) and 0.73 Å^–1^ (*n* = 1), corresponding to the (020) plane and the (040) plane of PEAI-induced 2D perovskite, presented in Figure S5b.^28,45^ For the OAI sample, the peak at *q* = 0.23 Å^–1^ indicates the (020) plane (*n* = 2) of the 2D perovskite as shown in Figure 3b.^46^ The BAI sample exhibits (020) and (040) plane peaks at *q* = 0.30 and 0.60 Å^–1^ (*n* = 2), as shown in Figures 3c and S4c.^47^ Moreover, as seen in Figure S5d, diffraction peaks corresponding to *n* = 3 are additionally observed at *q* = 0.27 and 0.74 Å^–1^, which are associated with the (020) and (060) planes of BAI-induced 2D perovskite, respectively.^48^ As the incident angle increases from 0.1° to 0.6°, allowing a deeper penetration of the X-ray beam below the top surface part of the 2D/3D perovskite heterojunction film, the intensity of the distinct diffraction peaks associated with the 2D perovskite gradually diminishes. Simultaneously, Debye–Scherrer diffraction rings corresponding to the bulk 3D perovskites become more prominent. This observation suggests that a highly oriented 2D RP phase perovskite layer is predominantly formed near the surface of the heterojunction structure. Organic spacer cations passivate undercoordinated Pb^2+^ ions at the film surface through coordination bonding, forming 2D layers at grain boundaries or surfaces that enhance film density, smoothness, and environmental stability. The combination of gas quenching with organic spacer cation passivation not only optimizes film formation kinetics and crystal uniformity but also actively reduces defect density, resulting in significantly improved film quality compared to the use of antisolvent methods alone.^31,32^

**Figure 3:**
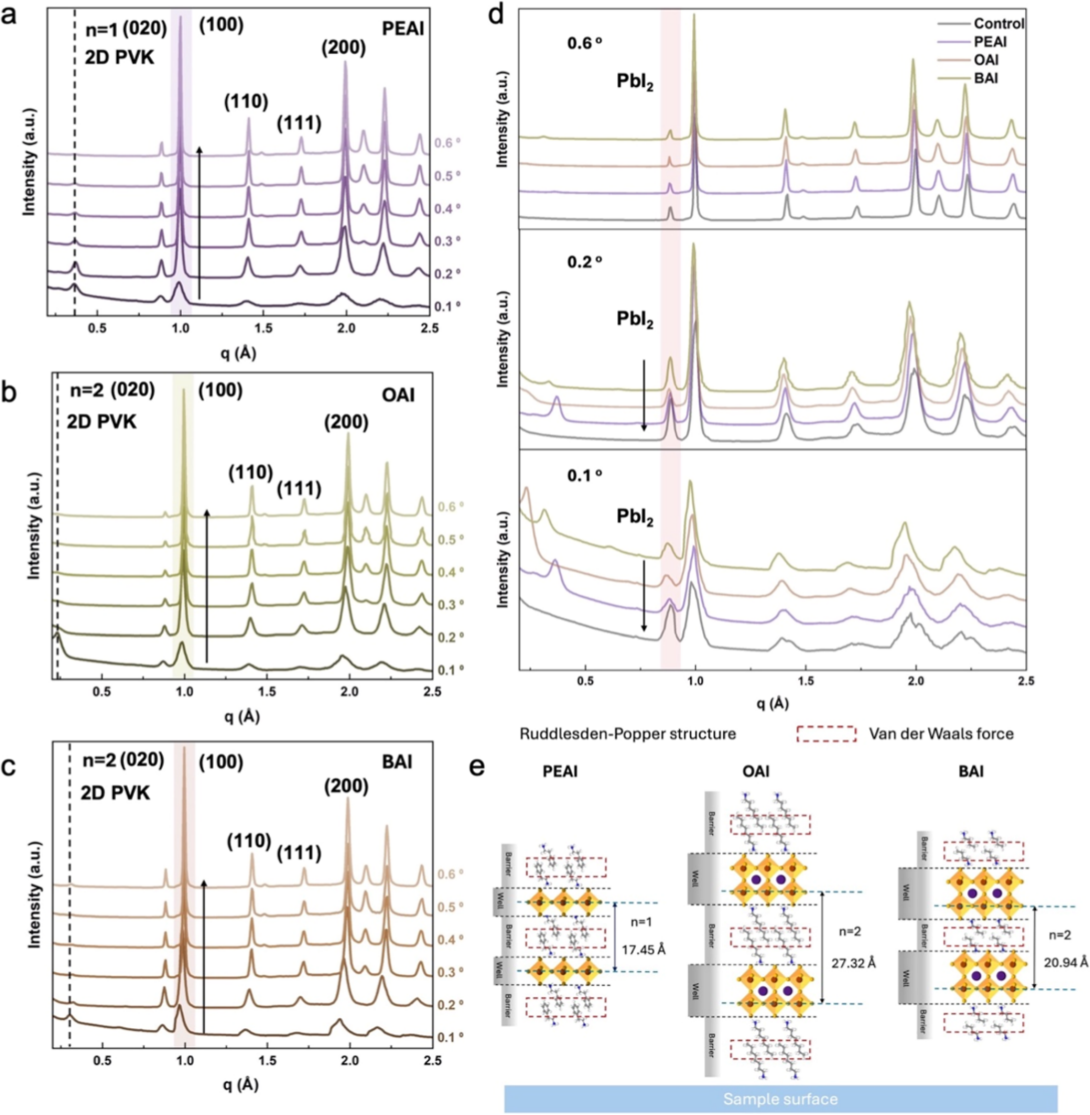
Pseudo XRD spectra (0.1°–0.6° incident angles) of perovskite films with (a) PEAI, (b) OAI, (c) BAI surface passivation. (d) Pseudo XRD spectra (0.1°, 0.2°, and 0.6° incident angles) of perovskite films with and without surface passivation. (e) Schematic representation of the *d-*spacing of the dominant crystallized plane and quantum well structure of a 2D perovskite layer parallel to the sample surface.

After passivation, the residual PbI_2_ on the surface of the 3D perovskite film can react with the lone pair electrons of the nitrogen atom in the terminal functional group of the organic spacer cation in the surface passivation materials to form a 2D perovskite. It is worth noting that the reduction of uncoordinated PbI_2_ through surface passivation remains rather limited.^23^ This is also evident in Figure 3d, where the intensity of the PbI_2_ diffraction peak decreases to a certain extent after surface passivation at incident angles of 0.1° and 0.2°, indicating changes in the 3D perovskite film surface region. In contrast, at an incident angle of 0.6°, the diffraction peak intensity of PbI_2_ remains nearly unchanged, suggesting minimal variation after passivation in the bulk region of the 3D perovskite, indicating that the passivation materials preferentially interact with the residual PbI_2_ on the surface and form interfacial 2D perovskite layers as mentioned before.

According to Figure 3e, the *d-*spacing (*d* = 2π/*q*) of the dominant 2D perovskites varies with the passivation materials. The corresponding *d-*spacing of the different *n*-value 2D perovskite of the PEAI, BAI, and OAI samples increases from 17.45 to 20.94 to 27.32 Å. This 2D perovskite layer acts as a quantum well, shown in Figure 3e, that passivates surface defects but also forms a barrier for charge carrier transport.^27,49^ The smaller *d-*spacing of PEA-based perovskite originates from the smallest cation size and π-conjugated benzene ring, which enables closest packing of the organic layers and strongest van der Waals and π–π interactions between adjacent layers, compared with OA and BA cation.^50,51^ This leads to the narrowest quantum well barriers, which reduces the energy barrier for hole transport across the 2D/3D interface.^52,53^ In combination with the face-on orientation, these contribute to the superior charge transport compared to BAI and OAI-passivated perovskite.

In Figure 3e, the rectangular mark highlights the van der Waals interactions among the organic cations in the 2D perovskite layer. The van der Waals force between organic spacer cations helps to stabilize the layered stacking structure of the 2D perovskite, promoting an ordered crystal arrangement.^54,55^ Regarding tunneling effects, it is possible that charge carriers may partially tunnel through very thin 2D perovskite layers, particularly when the layer thickness is on the order of a few nanometers.^56^ In the cross-sectional SEM images, the 2D layer is extremely thin and forms directly on top of the 3D perovskite, suggesting that quantum tunneling could contribute to charge carrier transport across the barrier.^57^ However, the overall extraction efficiency is likely influenced by a combination of factors, including barrier height (related to *d-*spacing), interfacial defect density, and charge carrier mobility within both the 2D and 3D phases.

### 2.3 Characterization of Perovskite Solar Cells

The photovoltaic devices are fabricated with a typical regular device layout consisting of ITO/SnO_2_/perovskite/surface passivation/spiro-OMeTAD/Au. The cross-sectional SEM images of all four types of PSCs are shown in Figure 4a. It is visible that the thickness of the 3D perovskite layer in the four devices ranges from 500 to 600 nm. Since the 2D perovskite layer grows directly on the surface of the 3D perovskite and has a thickness on the nanometer scale, it is beyond the spatial resolution of conventional cross-sectional SEM, making direct thickness determination impractical. After passivation, the surface of the 3D perovskite film becomes noticeably smoother. We investigate the impact of the passivation layer on the device performance and stability. The *J*–*V* curves of the best-performing solar cells with and without surface passivation are presented in Figure 4b. PCE values of the champion devices for the Control, PEAI, OAI, and BAI samples are 13.42%, 16.26%, 15.39%, and 14.57%, respectively. The results indicate that surface passivation leads to a certain degree of improvement in the PCE, with PEAI showing the most significant enhancement among the passivation treatments. In addition, the corresponding champion device *J*–*V* curves with forward and reverse scans are shown in Figure S6, showcasing the minor hysteresis.

**Figure 4:**
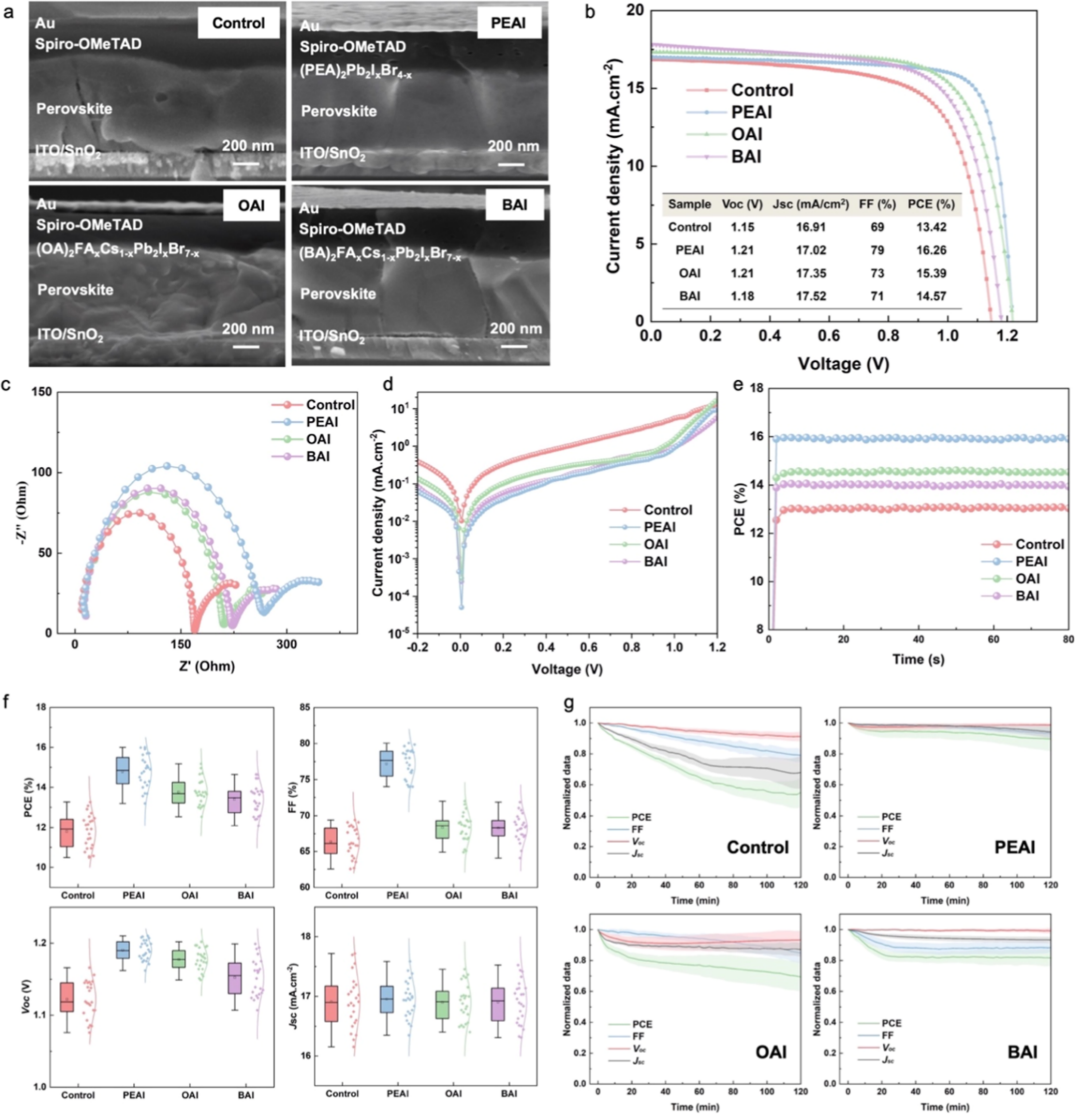
(a) SEM cross-sectional images, (b) champion device *J*–*V* curves, (c) electrochemical impedance spectroscopy (EIS) Nyquist plot under light illumination with *V*_oc_ bias, (d) dark state *J*–*V* curves, (e) stabilized power output (SPO), (f) photovoltaic parameters distribution, and (g) temporal evolution of PCE, *J*_sc_, *V*_oc_, and FF values after 120 min continuous illumination under a nitrogen atmosphere at room temperature of the solar cell device without surface passivation and devices with PEAI, OAI, and BAI passivation.

Despite the state-of-the-art PCEs of 1.77 eV, PSCs have achieved ∼21%, most of the devices are based on p-i-n configuration and processed in a controlled environment.^58,59^ In contrast, this work demonstrates the feasibility of processing perovskite films under ambient air conditions with ∼30–40% relative humidity, facilitating the transition from laboratory-scale fabrication to large-scale manufacturing. The observed efficiency gap between our work and the reported champion can reasonably be attributed to the combined effects of a device structure and ambient fabrication conditions. This comparison highlights the practical challenges of achieving high performance in an environment outside of an inert atmosphere.

According to the distribution of the PCE, fill factor (FF), short-circuit current (*J*_sc_), and open-circuit voltage (*V*_oc_) values shown in Figure 4f, the PEAI sample exhibits the highest PCE, FF, and *V*_oc_, demonstrating the most effective defect. This observation is in good agreement with the highest PL intensity and the widest bandgap of the PEAI sample, indicating less nonradiative charge carrier recombination caused by defects, resulting in an enhanced *V*_oc_, which is consistent with previous publications.^60–62^ This is also directly confirmed by the highest recombination resistance (*R*_rec_ ∼ 263 Ω) extracted from EIS analysis, together with the most ideal diode behavior observed in the dark *J*–*V* characteristics, as shown in Figure 4c,d.^63,64^ In comparison with insulating aliphatic amino cations in the OAI and BAI, aromatic amino cations in the PEAI-induced 2D perovskite achieve superior charge carrier transport capabilities. This arises from the presence of π-conjugated benzene rings in which delocalized π-electrons move freely along extended molecular orbitals.^65^ Additionally, strong intermolecular π–π interactions further facilitate the charge mobility. As a result, aromatic amino molecules typically offer enhanced electrical conductivity, leading to an excellent FF improvement of the PEAI sample.^66,67^ This interpretation is further corroborated by the lowest series resistance (*R*_s_ ∼ 3.7 Ω), as determined from the EIS results, as shown in Figure 4c. The resistance values for series and recombination derived from the Nyquist plot are shown in Table S1. Regarding the *J*_sc_, a loss or gain in light absorption typically results in a corresponding decrease or increase in *J*_sc_. As the passivation 2D perovskite layer is very thin on the top surface of the 3D perovskite, it cannot induce noticeable parasitic absorption losses.^49^ On the one hand, the low-n 2D perovskite organic spacer cations form a barrier that hinders charge carrier transport after surface passivation due to quantum confinement.^27^ On the other hand, the effective defect passivation provided by these low-n 2D perovskite layers significantly suppresses nonradiative recombination, enabling smoother charge carrier transport, especially the better transport due to π-conjugated benzene rings in the PEAI sample. The combined effect of quantum confinement, defect passivation, and high-quality interfaces enables an efficient charge carrier extraction without significantly compromising *J*_sc_.

The initial stable power output (SPO) tracking under maximum power point conditions provides a critical real-time assessment of the photocurrent hysteresis and the initial operational stability of the devices. As shown in Figure 4e, all four device configurations show flat and nondecaying SPO curves over the 80 s measurement period, indicating rapid stabilization and no significant initial performance degradation under the test conditions.

In addition, we monitor the operational stability of these devices under a nitrogen atmosphere and 120 min continuous illumination at room temperature, where the control, PEAI, OAI, and BAI samples retained 55.1%, 88%, 69.3%, and 81% of their initial PCE values, respectively, as shown in Figure 4g. Notably, a surface passivation treatment improves the device stability under continuous illumination. This finding can be attributed to surface defect passivation provided by the 2D perovskite layers. In addition, the reduced amount of residual PbI_2_ after surface passivation according to the GIWAXS results discussed earlier helps to suppress light-induced degradation by generating trap states.^68^ Among the four studied types of PSCs, the PEAI sample shows the most significant improvement in stability. This improvement results from a dense and rigid 2D perovskite layer as evidenced by the smaller *d-*spacing revealed in the earlier GIWAXS analysis, which formed after PEAI surface passivation, exhibiting strong defect passivation and efficient suppression of ion migration.^69^ Additionally, the strong van der Waals force between aromatic organic cations of PEAI contributes to a well-ordered 2D perovskite layer that minimizes light-induced ion redistribution while maintaining favorable charge carrier transport pathways.^54,55^ The π-conjugated benzene rings in the PEAI-induced 2D perovskite can help to improve film crystallinity and enhance interlayer interactions, resulting in a more robust and uniform 2D layer that mitigates ion migration and enhances structural integrity.^70^ Together, these factors, defect passivation, improved crystallinity, strong van der Waals interactions, and favorable interfacial properties, explain why PEAI provides the most effective stability enhancement. In contrast, the BAI sample with a more flexible alkyl chain forms a relatively loose 2D perovskite layer that provides moderate defect passivation but offers less effective ion migration suppression. This combination leads to a decline in performance during continuous illumination, placing its stability at an intermediate level. The OAI sample experiences a notable decay in *J*_sc_ and FF and exhibits the poorest stability. For the OAI sample, the long alkyl chain in the organic spacer cation of OAI and an induced *n* = 2 2D perovskite layer leads to the formation of wider quantum wells and barriers, which significantly hinder charge carrier extraction and further lead to charge accumulation.^27,30^ This finding suggests that while OAI effectively passivates surface defects, it likely has a detrimental effect on the interfacial charge carrier dynamics and ion migration due to the relatively wider OAI-induced 2D perovskite *d-*spacing, potentially introducing new transport barriers or altering interface dipoles, resulting in making the device more susceptible to interface degradation and nonradiative recombination under continuous illumination.

## 3 Conclusion

In summary, we have explored the optimal experimental parameters for gas quenching to fabricate wide-bandgap PSCs. Notably, this gas quenching method eliminates the use of antisolvents and is carried out entirely under ambient conditions, making the fabrication process more environmentally friendly and cost-effective, which together is an important step toward real-world use of PSCs. Furthermore, we develop a surface passivation strategy for these 3D FA_0_._8_Cs_0_._2_Pb­(I_0_._6_Br_0_._4_)_3_ perovskite films using PEAI, OAI, and BAI molecules. This approach leads to the formation of 2D perovskite layers with Ruddlesden–Popper (RP) structures and varying *n* values on the surface of 3D perovskite films, effectively reducing the defect density, improving the film morphology, and enhancing the device performance. As a result, devices with PEAI surface passivation achieve a champion PCE of 16.26% along with a high *V*_oc_ of 1.21 V, surpassing that of the control device (PCE = 13.42%, *V*_oc_ = 1.15 V). The optimized device with PEAI passivation maintains approximately 88% of its initial PCE after 120 min of continuous illumination under a nitrogen atmosphere at room temperature. Devices without surface passivation and those with OAI and BAI passivation obtain relatively lower champion PCE values of 13.42%, 15.39%, and 14.57%, respectively. They undergo light-induced degradation more easily than devices with PEAI passivation. These findings suggest that the introduction of aromatic amino molecules contributes to the development of more efficient and stable wide-bandgap perovskite solar cells. The demonstrated balance of efficiency, stability, and manufacturability achieved by the gas quenching method and surface passivation strategy paves the way for wide-bandgap perovskite solar cells in tandem solar cells, showing great potential for scalable production methods such as printing, which can achieve industrial scalability aligned with green manufacturing principles.

## 4 Experimental Section

### 4.1 Materials

Lead­(II) iodide (PbI_2_), lead bromide (PbBr_2_), formamidinium iodide (FAI), cesium iodide (CsI), chlorobenzene (CB), *N*,*N*-dimethylformaide (DMF), dimethyl sulfoxide (DMSO), deionized water (H_2_O), isopropanol (IPA), N^2^,N^2^,N^2′^,N^2′^,N,^7^N^7^,N^7′^,N^7′^-octakis­(4-methoxyphenyl)-9,9′-spirobi­[9*H*-fluorene]-2,2′,7,7′-tetramine (spiro-OMeTAD), lithium bis­(trifluoromethanesulfonyl)­imidate (Li-TFSI), 4-*tert*-butylpyridine (TBP), acetonitrile (ACN), *n*-octylammonium iodide (n-OAI), and *n*-butylammonium (*n*-BAI) were purchased from Sigma-Aldrich. Tin dioxide colloidal solution (SnO_2_) was purchased from Alfa Aesar. Phenethylammonium iodide (PEAI) was from Sigma-Aldrich. The patterned indium-doped tin oxide substrates (ITO, high transmittance, 2.5 × 2.5 cm^2^, 15 Ω/Sq) were purchased from Yingkou Shangneng Photoelectric Material Co., Ltd. Unless otherwise specified, all solvents and chemicals were used as received without further purification.

### 4.2 Regular (n-i-p) PSC Fabrication

ITO substrates were sequentially cleaned in deionized water, acetone, isopropanol, and ethanol using an ultrasonic bath for 30 min each. Before use, the substrates underwent a 10 min ozone treatment to clean the surface and enhance surface wettability. For the hole-blocking layer, the tin dioxide colloidal solution was diluted in deionized water with a volume ratio of 1:4 and then the SnO_2_ solution was spin-coated on the plasma-treated ITO substrate at 3000 rpm for 30 s. Subsequently, the substrates were annealed for 30 min at 150 °C. For the perovskite active layer, 1.35 M FA_0.8_Cs_0.2_Pb­(I_0.6_Br_0.4_)_3_ precursor solution was spin-coated on the SnO_2_ layer at 2000, 3000, and 4000 rpm for 40 s under ambient conditions. N_2_ flow with 2, 3, and 4 bar pressure was applied on the dynamic substrate 10 s after the start of spin coating. The perovskite film on the substrate turned dark brown at the end of gas quenching, followed by annealing at 150 °C for 15 min. For the surface passivation layer, 2 mg/mL of PEAI, OAI, and BAI in IPA were prepared and spin-coated onto the active layer at 5000 rpm for 30 s, followed by annealing at 100 °C for 5 min. For the electron-blocking layer, 17.5 μL of Li-TFSI solution (520 mg of Li-TFSI in 1 mL of ACN) and 28.8 μL of TBP were added to the spiro-OMeTAD solution (72.3 mg of spiro-OMeTAD in 1 mL CB). Then the spiro-OMeTAD solution was spin-coated onto the surface passivation layer at 5000 rpm for 30 s. Subsequently, the samples were placed in a sealed container with approximately 1% humidity and left overnight to undergo oxidation. For the top metal electrode, an 80 nm thick gold layer was deposited by thermal evaporation using a six-pixel substrate holder. During the *J*–*V* measurements, a nonreflective mask defining an active area of 0.08 cm^2^ was applied. In this study, the perovskite sample without surface passivation is labeled as Control; the samples with PEAI, OAI, and BAI surface passivation are labeled as PEAI, OAI, and BAI.

### 4.3 Characterization Methods

Scanning electron microscopy (SEM) was performed using Zeiss Gemini NVision 40 and ZEISS EVO MA10 systems. Atomic force microscopy (AFM) measurements were conducted by using a Nanosurf FlexAFM system under ambient conditions. XRD was performed using a Bruker D8 Advance with a motorized reflectometry stage with characteristic Cu K-alpha emission at λ = 1.5418 Å. A PerkinElmer Lambda 650S spectrometer was used in this work to carry out UV–vis spectroscopy measurements. The PL spectra were collected by a PerkinElmer LS 55 fluorescence spectrometer under a 45  nm excitation laser. The static *J*–*V* measurement was conducted under AM 1.5 G illumination (100 W/m^2^) by a solar simulator assembled with a Keithley 2611B source meter. The EIS was conducted under AM 1.5 G illumination (100 W/m^2^) by a solar simulator. The dark state *J*–*V* measurement was conducted by using a Keithley 2611B source meter. A Si reference cell (Fraunhofer ISE019-2015) was used to calibrate the light intensity of a solar simulator. The operational stability of the PSCs was measured under the illumination of 150 W Xenon short-arc lamps (PE150AF, Excelitas Technologies), with initial UV output (<390 nm, total output in all directions) of 0.9 W. Grazing-incidence wide-angle X-ray scattering (GIWAXS) measurements were performed at the P03/MiNaXS beamline of the PETRA III storage ring at DESY.^71^ Data were recorded using a LAMBDA 9 M detector (XSpectrum, Germany, pixel size 55 μm), with X-rays operated at a photon energy of 12 keV and a beam size of 29 × 21 μm^2^, corresponding to a wavelength of 1.033 Å. The sample-to-detector distance was maintained at 230.2 mm. Incident angles (α_i_) from 0.1° to 0.6° were selected to investigate the crystal structure of the perovskite films at different depths. The collected data were processed and analyzed using the INSIGHT software package.^72^

## Supplementary Material



## Data Availability

All relevant data can also be found at the following public repository: https://doi.org/10.14459/2025mp1839308

## Notes

SEM images, AFM images, and *J*–*V* curves of alternative fabrication parameters, 2D GIWAXS data at different incident angles, zoomed-in pseudo XRD data, champion device *J*–*V* curves, and resistance values (PDF)

## References

[1] Kojima A., Teshima K., Shirai Y., Miyasaka T. (2009). Organometal Halide
Perovskites as Visible-Light Sensitizers for Photovoltaic Cells. J. Am. Chem. Soc..

[2] Liu S., Li J., Xiao W., Chen R., Sun Z., Zhang Y., Lei X., Hu S., Kober-Czerny M., Wang J. (2024). Buried
Interface Molecular Hybrid for Inverted Perovskite Solar Cells. Nature.

[3] Jeon N. J., Noh J. H., Kim Y. C., Yang W. S., Ryu S., Seok S. I. (2014). Solvent Engineering for High-Performance Inorganic–Organic
Hybrid Perovskite Solar Cells. Nat. Mater..

[4] Jeon N. J., Noh J. H., Yang W. S., Kim Y. C., Ryu S., Seo J., Seok S. I. (2015). Compositional
Engineering of Perovskite Materials for
High-Performance Solar Cells. Nature.

[5] An Y., Zhang N., Zeng Z., Cai Y., Jiang W., Qi F., Ke L., Lin F. R., Tsang S.-W., Shi T. (2024). Optimizing Crystallization
in Wide-Bandgap Mixed Halide Perovskites
for High-Efficiency Solar Cells. Adv. Mater..

[6] Fang Z., Deng B., Jin Y., Yang L., Chen L., Zhong Y., Feng H., Yin Y., Liu K., Li Y. (2024). Surface Reconstruction of Wide-bandgap Perovskites
Enables Efficient Perovskite/Silicon Tandem Solar Cells. Nat. Commun..

[7] Mei J., Yan F. (2025). Recent Advances in
Wide-Bandgap Perovskite Solar Cells. Adv. Mater..

[8] Choi H., Choi K., Choi Y., Kim T., Lim S., Park T. (2020). A Review on Reducing Grain Boundaries and Morphological Improvement
of Perovskite Solar Cells from Methodology and Material-Based Perspectives. Small Methods.

[9] Wu Z., Sang S., Zheng J., Gao Q., Huang B., Li F., Sun K., Chen S. (2024). Crystallization
Kinetics of Hybrid
Perovskite Solar Cells. Angew. Chem., Int. Ed..

[10] Sun K., Guo R., Liang Y., Heger J. E., Liu S., Yin S., Reus M. A., Spanier L. V., Deschler F., Bernstorff S. (2023). Morphological Insights into the Degradation of Perovskite Solar Cells
under Light and Humidity. ACS Appl. Mater. Interfaces.

[11] Taylor A. D., Sun Q., Goetz K. P., An Q., Schramm T., Hofstetter Y., Litterst M., Paulus F., Vaynzof Y. (2021). A General Approach
to High-efficiency Perovskite Solar Cells by Any Antisolvent. Nat. Commun..

[12] Ying Z., Su S., Li X., Chen G., Lian C., Lu D., Zhang M., Guo X., Tian H., Sun Y. (2025). Antisolvent Seeding
of Self-assembled Monolayers for Flexible Monolithic
Perovskite/Cu­(In,Ga)­Se_2_ Tandem Solar Cells. Nat. Energy.

[13] Ghosh S., Mishra S., Singh T. (2020). Antisolvents in Perovskite
Solar
Cells: Importance, Issues, and Alternatives. Adv. Mater. Interfaces.

[14] Huang F., Dkhissi Y., Huang W., Xiao M., Benesperi I., Rubanov S., Zhu Y., Lin X., Jiang L., Zhou Y. (2014). Gas-assisted Preparation of Lead Iodide Perovskite
Films Consisting of A Monolayer of Single Crystalline Grains for High
Efficiency Planar Solar Cells. Nano Energy.

[15] Conings B., Babayigit A., Klug M. T., Bai S., Gauquelin N., Sakai N., Wang J. T.-W., Verbeeck J., Boyen H.-G., Snaith H. J. (2016). A Universal Deposition Protocol for Planar Heterojunction
Solar Cells with High Efficiency Based on Hybrid Lead Halide Perovskite
Families. Adv. Mater..

[16] Jiang Q., Tong J., Scheidt R. A., Wang X., Louks A. E., Xian Y., Tirawat R., Palmstrom A. F., Hautzinger M. P., Harvey S. P. (2022). Compositional Texture
Engineering for Highly Stable Wide-bandgap Perovskite Solar Cells. Science.

[17] Shi Z., Zhou J., Ma Y., Wei X., Wen T., Sun J., Cheng Z., Zhang M., Liu F., Yang S. (2025). Gas-Quenching Assisted Crystallization of Wide-Bandgap Perovskite
for Triple-Junction Perovskite Solar Cells. Small Methods.

[18] Lu H., Liu Y., Ahlawat P., Mishra A., Tress W. R., Eickemeyer F. T., Yang Y., Fu F., Wang Z., Avalos C. E. (2020). Vapor-assisted Deposition of Highly Efficient,
Stable Black-phase
FAPbI_3_ Perovskite Solar Cells. Science.

[19] Rujisamphan N., Soe K. T., Moontragoon P., Yaro A. A., Ketsombun E., Supruangnet R., Luo D., Huang Z.-H., Liu S.-W., Supasai T. (2025). Two-Dimensional Surface
Passivation with Distinct Cations
Enabling High Efficiency and Mechanical Durability in Flexible Perovskite
Solar Cells. ChemSusChem.

[20] Fu Q., Xu Z., Tang X., Liu T., Dong X., Zhang X., Zheng N., Xie Z., Liu Y. (2021). Multifunctional
Two-Dimensional
Conjugated Materials for Dopant-Free Perovskite Solar Cells with Efficiency
Exceeding 22%. ACS Energy Lett..

[21] He Q., Worku M., Liu H., Lochner E., Robb A. J., Lteif S., Vellore
Winfred J. S.
R., Hanson K., Schlenoff J. B., Kim B. J. (2021). Highly Efficient and
Stable Perovskite Solar Cells Enabled by Low-Cost Industrial Organic
Pigment Coating. Angew. Chem., Int. Ed..

[22] Jiang X., Zeng J., Sun K., Li Z., Xu Z., Pan G., Guo R., Liang S., Bulut Y., Sochor B. (2024). Sputter-deposited TiO_x_ Thin Film as A Buried Interface
Modification Layer for Efficient and Stable Perovskite Solar Cells. Nano Energy.

[23] Wu G., Liang R., Ge M., Sun G., Zhang Y., Xing G. (2022). Surface Passivation Using 2D Perovskites
toward Efficient and Stable
Perovskite Solar Cells. Adv. Mater..

[24] Jiang Q., Zhao Y., Zhang X., Yang X., Chen Y., Chu Z., Ye Q., Li X., Yin Z., You J. (2019). Surface Passivation
of Perovskite Film for Efficient Solar Cells. Nat. Photonics.

[25] Rahil M., Ansari R. M., Prakash C., Islam S. S., Dixit A., Ahmad S. (2022). Ruddlesden–Popper 2D Perovskites
of Type (C_6_H_9_C_2_H_4_NH_3_)_2_(CH_3_NH_3_)_n–1_Pb_n_I_3n+1_ (n = 1– 4) for Optoelectronic
Applications. Sci. Rep..

[26] Sun K., Guo R., Liu S., Guo D., Jiang X., Huber L. F., Liang Y., Reus M. A., Li Z., Guan T. (2024). Deciphering Structure and Charge Carrier Behavior in
Reduced-Dimensional
Perovskites. Adv. Funct. Mater..

[27] Peng S., Ma J., Li P., Zang S., Zhang Y., Song Y. (2022). Regulation
of Quantum Wells Width Distribution in 2D Perovskite Films for Photovoltaic
Application. Adv. Funct. Mater..

[28] Chen R., Gu L., Su J., Feng Y., Deng H., Zhang J., Bao Y., Wang D., Song X., Zhao L. (2025). Stabilizing
2D Perovskite Passivation Layer with Mixed Spacer Cations for Efficient
and Stable Perovskite Solar Cells. Nano Energy.

[29] Xiang L., Cao Y., Sun J., Li D., Liu H., Gao F., Chen C., Li S. (2024). π-Conjugated
Aromatic Amino
Molecule for Interfacial Modification in High-Performance Perovskite
Solar Cells. Surf. Interfaces.

[30] Kim H., Lee S.-U., Lee D. Y., Paik M. J., Na H., Lee J., Seok S. I. (2019). Optimal Interfacial Engineering with Different Length
of Alkylammonium Halide for Efficient and Stable Perovskite Solar
Cells. Adv. Energy Mater..

[31] Gou Y., Tang S., Yuan C., Zhao P., Chen J., Yu H. (2024). Research Progress of Green Antisolvent for Perovskite Solar Cells. Mater. Horiz..

[32] Azhar M., Cuzzupè D. T., Yalcinkaya Y., Haider M. I., Schütz E. R., Schupp S. M., Temitmie Y. A., Hooijer R., Aydin E., Schmidt-Mende L. (2025). Impact of Antisolvent and Gas Quenching on Wrinkling
in Cs0.15FA0.85Pb­(I0.6Br0.4)­3 Perovskite Films. ACS Appl. Mater. Interfaces.

[33] Majewski M., Qiu S., Ronsin O., Lüer L., Le Corre V. M., Du T., Brabec C. J., Egelhaaf H. J., Harting J. (2025). Simulation of Perovskite
Thin Layer Crystallization with Varying Evaporation Rates. Mater. Horiz..

[34] Zhang Y., Wei X., Yu B., Zeng R., Kan L., Tang T., Yu H. (2025). Residual PbI_2_ Conversion and Crystallization Control for
Ambient-Air Fabrication of Industrially Viable Perovskite Solar Cells. Adv. Funct. Mater..

[35] Azhar M., Yalcinkaya Y., Cuzzupè D. T., Abebe Temitmie Y., Haider M. I., Schmidt-Mende L. (2025). Perovskite
Thin Films Solar Cells:
The Gas Quenching Method. Materials and Sustainability.

[36] Peng W., Wei J., Li H., Feng W., Liu M., Xu T., Qiu S., Liu C., Wagner M., Distler A. (2025). Balancing
the Supersaturation Rate and Coordination Capability for Upscaling
High-Performance Perovskite Photovoltaics. Energy
Environ. Sci..

[37] Kim J., Ho-Baillie A., Huang S. (2019). Review of Novel Passivation Techniques
for Efficient and Stable Perovskite Solar Cells. Sol. RRL.

[38] Jiang X., Chen S., Li Y., Zhang L., Shen N., Zhang G., Du J., Fu N., Xu B. (2021). Direct Surface
Passivation of Perovskite Film by 4-Fluorophenethylammonium Iodide
toward Stable and Efficient Perovskite Solar Cells. ACS Appl. Mater. Interfaces.

[39] Zou Y., Cui Y., Wang H.-Y., Cai Q., Mu C., Zhang J.-P. (2019). Highly
Efficient and Stable 2D–3D Perovskite Solar Cells Fabricated
by Interfacial Modification. Nanotechnology.

[40] Liu Y., Akin S., Pan L., Uchida R., Arora N., Milić J. V., Hinderhofer A., Schreiber F., Uhl A. R., Zakeeruddin S. M. (2019). Ultra Hydrophobic 3D/2D
Fluoroarene Bilayer-based Water-resistant Perovskite Solar Cells with
Efficiencies Exceeding 22%. Sci. Adv..

[41] Wang F., Bai S., Tress W., Hagfeldt A., Gao F. (2018). Defects Engineering
for High-Performance Perovskite Solar Cells. npj Flexible Electron..

[42] Ma Z., Dong Y., Wang R., Xu Z., Li M., Tan Z. a. (2023). Transparent Recombination Electrode
with Dual-Functional
Transport and Protective Layer for Efficient and Stable Monolithic
Perovskite/Organic Tandem Solar Cells. Adv.
Mater..

[43] Kaur N., Madan J., Perumal A., Pandey R. (2025). Current Matched All
Perovskite Tandem Solar Cells with Low Lead Perovskites Achieving
31.9% Efficiency and Enhanced Stability. Sci.
Rep..

[44] Wu S., Zhang J., Li Z., Liu D., Qin M., Cheung S. H., Lu X., Lei D., So S. K., Zhu Z. (2020). Modulation of Defects
and Interfaces through Alkylammonium
Interlayer for Efficient Inverted Perovskite Solar Cells. Joule.

[45] Seid B. A., Sarisozen S., Peña-Camargo F., Ozen S., Gutierrez-Partida E., Solano E., Steele J. A., Stolterfoht M., Neher D., Lang F. (2024). Understanding and Mitigating Atomic
Oxygen-Induced Degradation of Perovskite Solar Cells for Near-Earth
Space Applications. Small.

[46] Li F., Xie Y., Hu Y., Long M., Zhang Y., Xu J., Qin M., Lu X., Liu M. (2020). Effects of Alkyl Chain Length on
Crystal Growth and Oxidation Process of Two-Dimensional Tin Halide
Perovskites. ACS Energy Lett..

[47] Yang G., Ren Z., Liu K., Qin M., Deng W., Zhang H., Wang H., Liang J., Ye F., Liang Q. (2021). Stable and Low-photovoltage-loss Perovskite
Solar cells by Multifunctional
Passivation. Nat. Photonics.

[48] Stoumpos C. C., Cao D. H., Clark D. J., Young J., Rondinelli J. M., Jang J. I., Hupp J. T., Kanatzidis M. G. (2016). Ruddlesden–Popper
Hybrid Lead Iodide Perovskite 2D Homologous Semiconductors. Chem. Mater..

[49] Herterich J., Baretzky C., Unmüssig M., Maheu C., Glissmann N., Gutekunst J., Loukeris G., Mayer T., Kohlstädt M., Hofmann J. P. (2022). Toward Understanding the Short-Circuit Current
Loss in Perovskite Solar Cells with 2D Passivation Layers. Sol. RRL.

[50] Soe C. M. M., Stoumpos C. C., Kepenekian M., Traoré B., Tsai H., Nie W., Wang B., Katan C., Seshadri R., Mohite A. D. (2017). New
Type of 2D Perovskites
with Alternating Cations in the Interlayer Space, (C­(NH_2_)_3_)­(CH_3_NH_3_)_n_PbnI_3n+1_: Structure, Properties, and Photovoltaic Performance. J. Am. Chem. Soc..

[51] Quan L. N., Yuan M., Comin R., Voznyy O., Beauregard E. M., Hoogland S., Buin A., Kirmani A. R., Zhao K., Amassian A. (2016). Ligand-Stabilized Reduced-Dimensionality Perovskites. J. Am. Chem. Soc..

[52] Smith I. C., Hoke E. T., Solis-Ibarra D., McGehee M. D., Karunadasa H. I. (2014). A Layered
Hybrid Perovskite Solar-Cell Absorber with Enhanced Moisture Stability. Angew. Chem., Int. Ed..

[53] Li Y., Ding B., Chu Q.-Q., Yang G.-J., Wang M., Li C.-X., Li C.-J. (2017). Ultra-high Open-circuit Voltage of
Perovskite Solar Cells Induced by Nucleation Thermodynamics on Rough
Substrates. Sci. Rep..

[54] Ribeiro I. C., Moraes P. I. R., Bittencourt A. F. B., Da Silva J. L. F. (2024). Unveiling the
Impact of Organic Cation Passivation on Structural and Optoelectronic
Properties of Two-dimensional Perovskites Thin Films. Appl. Surf. Sci..

[55] Gao Y., Dong X., Liu Y. (2023). Recent Progress
of Layered Perovskite
Solar Cells Incorporating Aromatic Spacers. Nano-Micro Lett..

[56] Leng K., Wang L., Shao Y., Abdelwahab I., Grinblat G., Verzhbitskiy I., Li R., Cai Y., Chi X., Fu W. (2020). Electron
Tunneling at the Molecularly Thin
2D Perovskite and Graphene Van Der Waals Interface. Nat. Commun..

[57] Ma K., Hsu S.-N., Gao Y., Wei Z., Jin L., Finkenauer B. P., Huang L., Boudouris B. W., Mei J., Dou L. (2021). Organic Cation Engineering for Vertical Charge Transport
in Lead-Free Perovskite Quantum Wells. Small
Sci..

[58] Lian X., Jin M., Dai W., Lv Y., Luo M., Hu Y., Wang Z., Li H., Xu C., Jiang D. (2025). A Supramolecular Approach to Improve the Performance
and Operational
Stability of All-perovskite Tandem Solar Cells. Nat. Commun..

[59] Liu Z., Lin R., Wei M., Yin M., Wu P., Li M., Li L., Wang Y., Chen G., Carnevali V. (2025). All-perovskite Tandem
Solar Cells Achieving > 29% Efficiency with
Improved (100) Orientation in Wide-bandgap Perovskites. Nat. Mater..

[60] Hu Y., Schlipf J., Wussler M., Petrus M. L., Jaegermann W., Bein T., Müller-Buschbaum P., Docampo P. (2016). Hybrid Perovskite/Perovskite
Heterojunction Solar Cells. ACS Nano.

[61] Juarez-Perez E. J., Wuβler M., Fabregat-Santiago F., Lakus-Wollny K., Mankel E., Mayer T., Jaegermann W., Mora-Sero I. (2014). Role of the Selective Contacts in
the Performance of
Lead Halide Perovskite Solar Cells. J. Phys.
Chem. Lett..

[62] Zhang Y., Liu M., Eperon G. E., Leijtens T. C., McMeekin D., Saliba M., Zhang W., de Bastiani M., Petrozza A., Herz L. M. (2015). Charge
Selective Contacts, Mobile Ions and Anomalous Hysteresis in
Organic–Inorganic Perovskite Solar Cells. Mater. Horiz..

[63] Roose B., Dey K., Fitzsimmons M. R., Chiang Y.-H., Cameron P. J., Stranks S. D. (2024). Electrochemical
Impedance Spectroscopy of All-Perovskite
Tandem Solar Cells. ACS Energy Lett..

[64] Liu W., Sun W., Wang K., Xu H., Huo X., Yin R., Sun Y., Ji S., You T., Li W. (2023). Surface
Passivation of CsPbI_3_ Films for Efficient and Stable Hole-Transporting
Layer-Free Carbon-Based Perovskite Solar Cells. ACS Appl. Energy Mater..

[65] Zhuang J., Mao P., Luan Y., Yi X., Tu Z., Zhang Y., Yi Y., Wei Y., Chen N., Lin T. (2019). Interfacial
Passivation for Perovskite Solar Cells: The Effects of the Functional
Group in Phenethylammonium Iodide. ACS Energy
Lett..

[66] Lao Y., Yang S., Yu W., Guo H., Zou Y., Chen Z., Xiao L. (2022). Multifunctional
π-Conjugated
Additives for Halide Perovskite. Advanced Science.

[67] Ma K., Atapattu H. R., Zhao Q., Gao Y., Finkenauer B. P., Wang K., Chen K., Park S. M., Coffey A. H., Zhu C. (2021). Multifunctional Conjugated Ligand Engineering for Stable
and Efficient Perovskite Solar Cells. Adv. Mater..

[68] Tumen-Ulzii G., Qin C., Klotz D., Leyden M. R., Wang P., Auffray M., Fujihara T., Matsushima T., Lee J.-W., Lee S.-J. (2020). Detrimental Effect of
Unreacted PbI_2_ on the Long-Term
Stability of Perovskite Solar Cells. Adv. Mater..

[69] Cao R., Sun K., Liu C., Mao Y., Guo W., Ouyang P., Meng Y., Tian R., Xie L., Lü X. (2024). Structurally Flexible 2D Spacer for Suppressing
the Electron–Phonon
Coupling Induced Non-Radiative Decay in Perovskite Solar Cells. Nano-Micro Lett..

[70] Zhong H., You S., Wu J., Zhu Z.-K., Yu P., Li H., Wu Z.-Y., Li Y., Guan Q., Dai H. (2024). Multiple Interlayer
Interactions Enable Highly Stable X-ray Detection
in 2D Hybrid Perovskites. JACS Au.

[71] Buffet A., Rothkirch A., Döhrmann R., Körstgens V., Abul Kashem M. M., Perlich J., Herzog G., Schwartzkopf M., Gehrke R., Müller-Buschbaum P. (2012). P03, the
Microfocus and Nanofocus X-ray Scattering (MiNaXS) Beamline of the
PETRA III Storage Ring: the Microfocus Endstation. J. Synchrotron Radiat..

[72] Reus M. A., Reb L. K., Kosbahn D. P., Roth S. V., Müller-Buschbaum P. (2024). INSIGHT: In
Situ Heuristic Tool for the Efficient Reduction of Grazing-incidence
X-ray Scattering Data. J. Appl. Crystallogr..

