# Supplementary material for: Gas Quenching under Ambient Conditions for Efficient and Stable Wide-Bandgap Perovskite Solar Cells with Surface Passivation
Source: ACS Appl Mater Interfaces. 2025 Dec 31;18(1):1702–13. doi: 10.1021/acsami.5c21175 (PMC12781052; doi:10.1021/acsami.5c21175)
Supplement: Supplementary file 1 [file am5c21175_si_001.pdf]

Supporting Information

Gas quenching under ambient conditions for efficient and stable wide-bandgap perovskite solar cells with surface passivation

Zhaonan Jin [1], Xiongzhuo Jiang [1], Zerui Li [1], Xiaojing Ci [1], Guangjiu Pan [1], Lixing Li [1], Jinsheng Zhang [1], Xinyu Jiang [2], Sarathlal Koyiloth Vayalil [2, 3], Kun Sun [4], Stephan V. Roth [2, 5], and Peter Müller-Buschbaum [1,]*

1. Technical University of Munich, TUM School of Natural Sciences, Department of Physics, Chair for Functional Materials, James-Franck-Str. 1, 85748 Garching, Germany

2. Deutsches Elektronen-Synchrotron DESY, Notkestrasse 85, 22607 Hamburg, Germany

3. Department of Physics, Applied Science Cluster, UPES, 248007 Dehradun, India

4. Helmholtz-Zentrum Berlin für Materialien und Energie GmbH (HZB), Department Perovskite Tandem Solar Cells, Kekuléstr. 5, 12489 Berlin, Germany

5. KTH Royal Institute of Technology, Department of Fibre and Polymer Technology, Teknikringen 56-58, Stockholm SE-11428, Sweden

Corresponding Author:* Peter Müller-Buschbaum muellerb@ph.tum.de

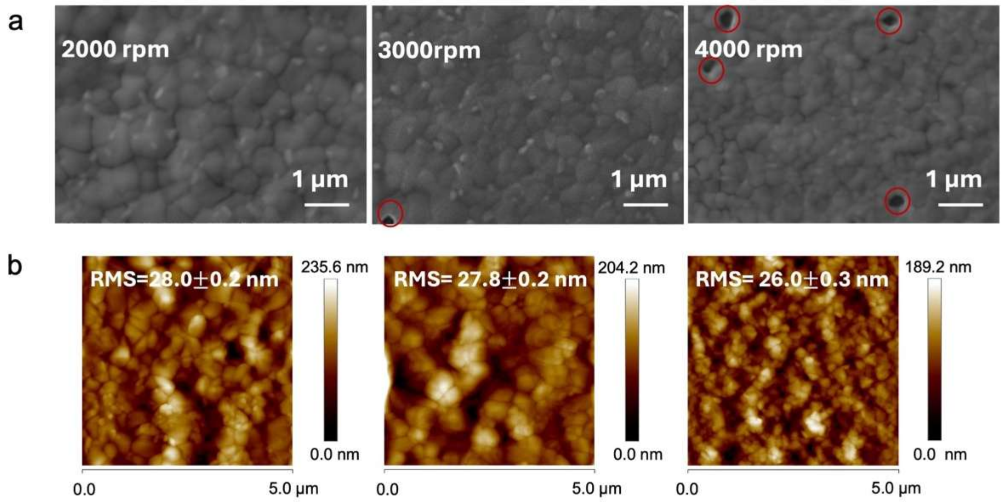

Figure S1. a) Top-view scanning electron microscope (SEM) images, and b) atomic force microscopy (AFM) topography images of perovskite films fabricated by gas quenching with 2000 rpm, 3000 rpm, and 4000 rpm spin coating speeds (from left to right) at a fixed gas flow pressure of 2 bar. Surface defects are indicated by red circles.

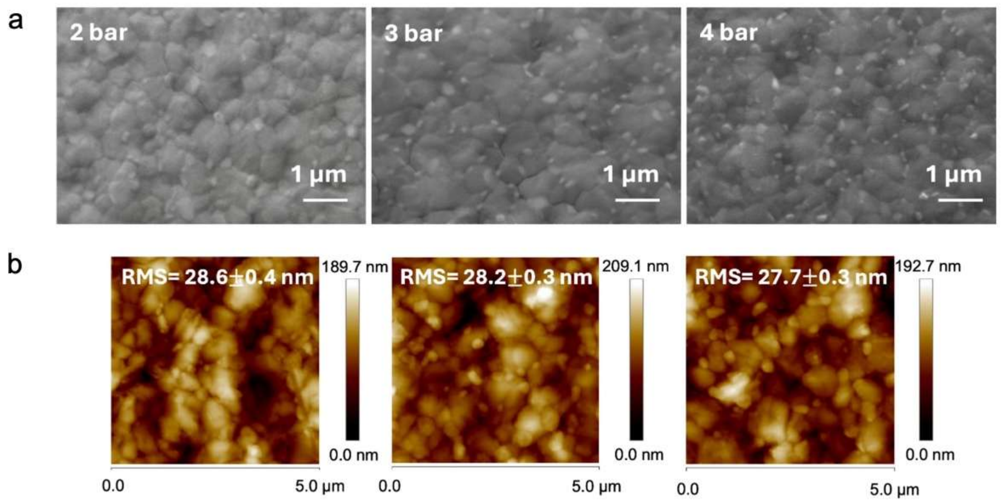

Figure S2. a) Top-view scanning electron microscope (SEM) images, and b) atomic force microscopy (AFM) topography images of perovskite films fabricated by gas quenching with 2 bar, 3 bar, and 4 bar gas flow pressure (from left to right) at a fixed spin coating speed of 2000 rpm.

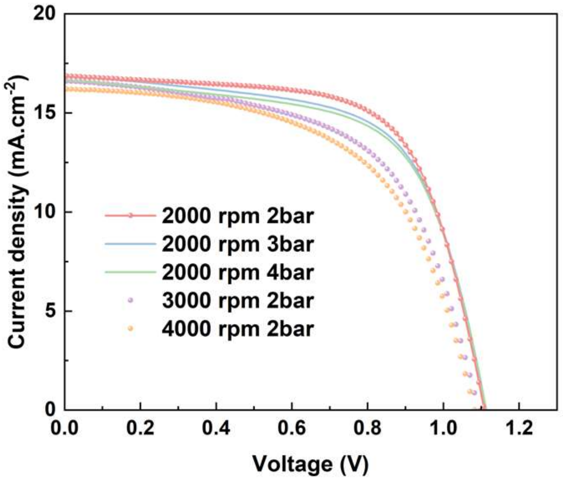

Figure S3. Static J-V curves of devices using different gas quenching parameters.

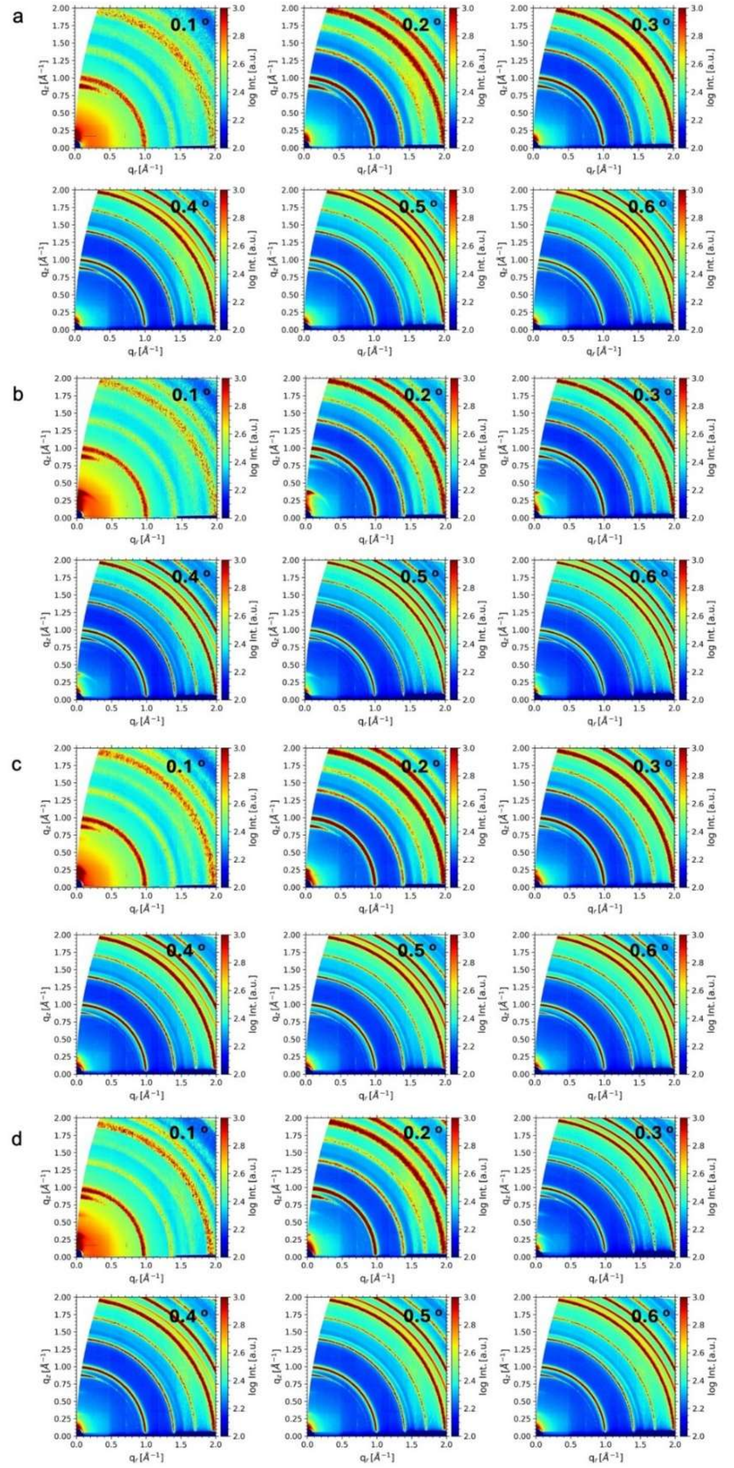

Figure S4. 2D GIWAXS data (0.1 º ~ 0.6 º incident angles) of a) the pristine perovskite film without surface passivation and the films with b) PEAI, c) OAI, and d) BAI passivation.

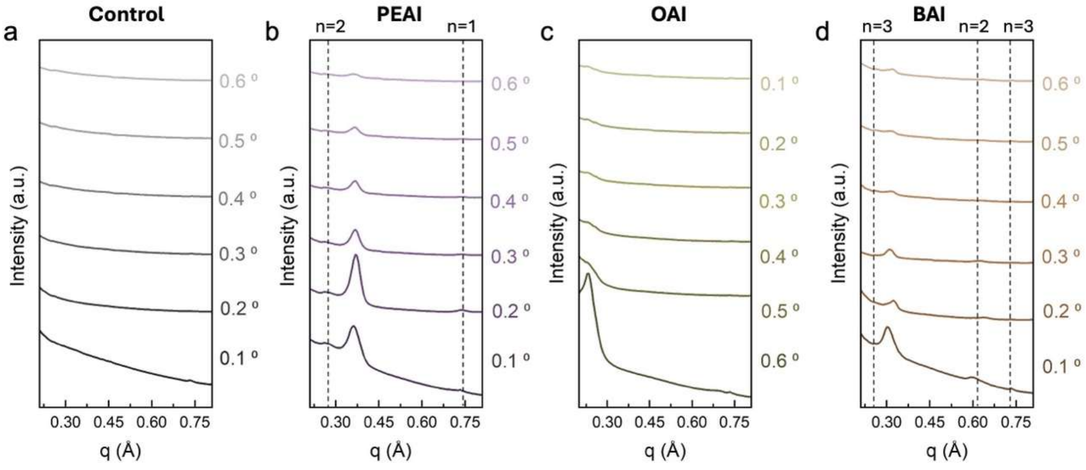

Figure S5. Zoomed-in pseudo XRD data (0.1 º ~ 0.6 º incident angles) of a) the pristine perovskite film without surface passivation and the films with b) PEAI, c) OAI and d) BAI passivation.

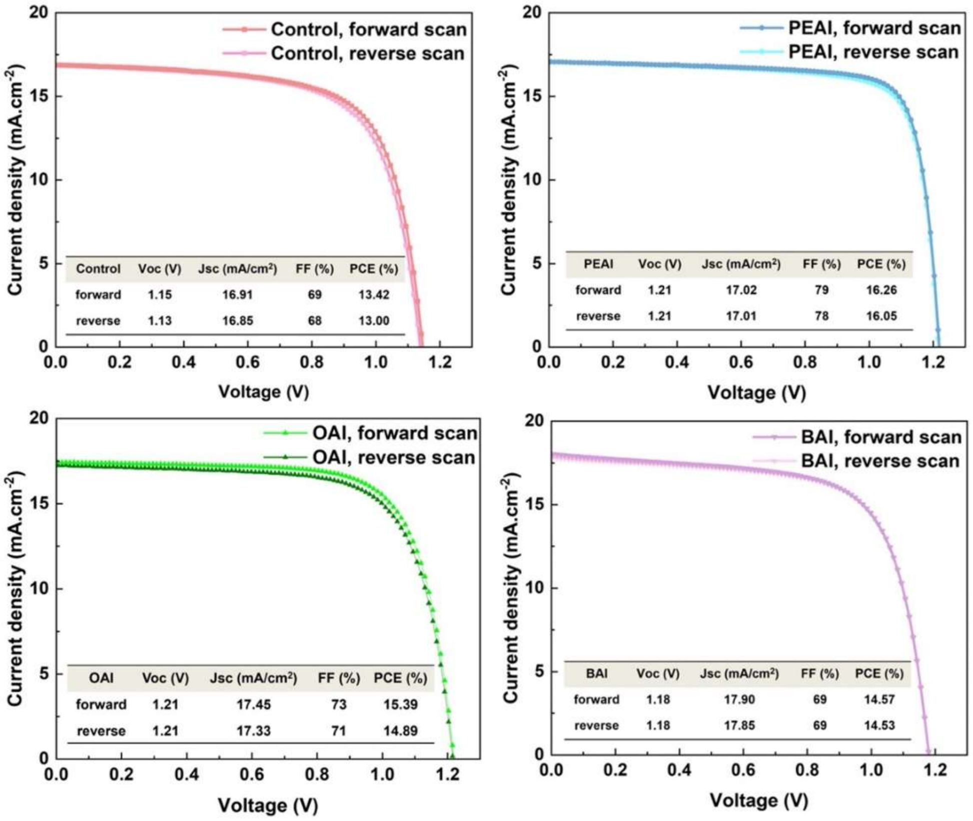

Figure S6. Champion device J-V curves with forward and reverse scans of the solar cell device without surface passivation and devices with PEAI, OAI, and BAI passivation.

	Control	PEAI	OAI	BAI
R_s (Ohm)	9.5	3.6	8.4	7.5
R_{rec} (Ohm)	161.2	262.7	200.8	214.4

Table S1. Values for series and recombination resistances derived from the Nyquist plot analysis.
